# Optical coherence tomography-derived macrophage arc as a novel biomarker for predicting adverse cardiovascular events in coronary artery disease: a multicentre study

**DOI:** 10.1093/ehjimp/qyag107

**Published:** 2026-06-12

**Authors:** Zelin Ou, Li Xie, Han Xiao, Bin Cui, Ke Wang, Yili Ma, Maoyu Zhao, Youzhu Qiu, Yao Zhang, Zhihui Zhang, Dehui Qian, Bin Liu, Xiaohui Zhao

**Affiliations:** Department of Cardiology, Xinqiao Hospital, Army Medical University, No.183 Xinqiao Avenue, Shapingba District, Chongqing, 400037, China; Department of Cardiology, Xinqiao Hospital, Army Medical University, No.183 Xinqiao Avenue, Shapingba District, Chongqing, 400037, China; Department of Cardiology, Xinqiao Hospital, Army Medical University, No.183 Xinqiao Avenue, Shapingba District, Chongqing, 400037, China; Department of Cardiology, Xinqiao Hospital, Army Medical University, No.183 Xinqiao Avenue, Shapingba District, Chongqing, 400037, China; Department of Cardiology, Xinqiao Hospital, Army Medical University, No.183 Xinqiao Avenue, Shapingba District, Chongqing, 400037, China; Department of Cardiology, Xinqiao Hospital, Army Medical University, No.183 Xinqiao Avenue, Shapingba District, Chongqing, 400037, China; Department of Cardiology, Xinqiao Hospital, Army Medical University, No.183 Xinqiao Avenue, Shapingba District, Chongqing, 400037, China; Department of Cardiology, Xinqiao Hospital, Army Medical University, No.183 Xinqiao Avenue, Shapingba District, Chongqing, 400037, China; Department of Statistics, Army Medical University, No.30 Gaotanyan Main Street, Shapingba District, Chongqing, 400038, China; Department of Cardiovascular Medicine, Center for Circadian Metabolism and Cardiovascular Disease, Southwest Hospital, Army Medical University, No.30 Gaotanyan Main Street, Shapingba District, Chongqing, 400038, China; Key Laboratory of Geriatric Cardiovascular and Cerebrovascular Disease, Ministry of Education of China, Chongqing, China; Department of Cardiology, Xinqiao Hospital, Army Medical University, No.183 Xinqiao Avenue, Shapingba District, Chongqing, 400037, China; Department of Cardiology, Secondary Hospital, Jilin University, No.218 Xiantai Street, Chaoyang District, Changchun 130041, Jilin Province, China; Department of Geriatrics, Xinqiao Hospital, Army Medical University, No.183 Xinqiao Avenue, Shapingba District, Chongqing, 400037, China

**Keywords:** macrophage arc, prognosis, optical coherence tomography, thin-cap fibroatheroma

## Abstract

**Aims:**

Macrophages play a crucial role in coronary artery plaque development and can be quantified as circumferential arc features via optical coherence tomography (OCT). However, the prognostic implications of macrophage arc characteristics remain underexplored.

**Methods and results:**

In this multicentre, retrospective study, consecutive patients with coronary artery disease (CAD) undergoing OCT between January 2017 and April 2023 were enrolled. The macrophage arc was evaluated using maximum arc, mean arc, and mean arc score (MAS) in the target vessel. Among 1025 patients (1173 vessels), 61 (5.9%) experienced major adverse cardiovascular events (MACEs). Receiver operating characteristic analysis identified optimal predictive thresholds: maximum arc ≥ 157.5°, mean arc ≥ 97.88°, and MAS ≥ 2.27 (all *P* < 0.001). Elevated mean arc [hazard ratio (HR) = 7.628, *P* < 0.0001], maximum arc (HR = 6.902, *P* < 0.0001), and MAS (HR = 6.704, *P* < 0.0001) were independently associated with MACEs. When combined with thin-cap fibroatheroma (TCFA) status, these parameters demonstrated enhanced predictive power: mean arc ≥ 97.88° + TCFA (HR = 8.779, *P* < 0.0001), maximum arc ≥ 157.5° + TCFA (HR = 8.149, *P* < 0.0001), and MAS ≥ 2.27 + TCFA (HR = 7.509, *P* < 0.0001). Notably, among TCFA-negative patients, a mean arc ≥ 97.88° showed markedly improved predictive capacity for MACEs (HR = 6.685, *P* < 0.001), as did maximum arc ≥ 157.5° (HR = 4.490, *P* < 0.001) and MAS ≥ 2.27 (HR = 5.126, *P* < 0.001).

**Conclusion:**

Macrophage arc parameters are strongly associated with long-term cardiovascular risk, serving as novel OCT-derived biomarkers for patients with CAD.

## Graphical Abstract

**Graphical Abstract:**
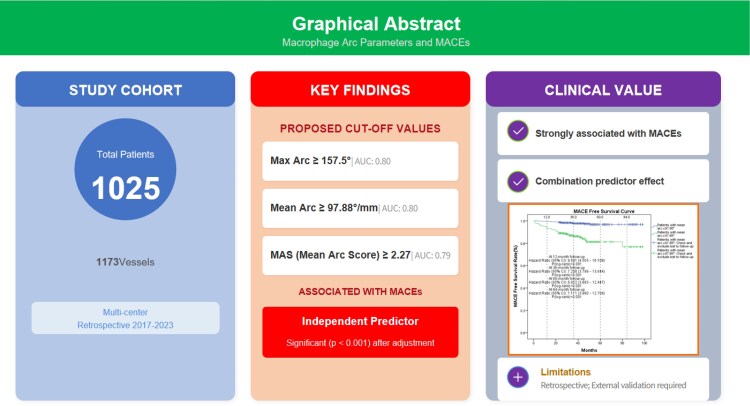
This graphical abstract summarizes a 2017–2023 multicenter retrospective cohort of 1025 patients and 1173 coronary vessels. Three OCT-derived macrophage arc metrics (maximum arc, mean arc, mean arc score [MAS]) are independent predictors of major adverse cardiovascular events (MACEs), with optimized cutoffs and good AUC values validated by adjusted multivariate analysis (all p < 0.001). Kaplan–Meier curves illustrate their prognostic value for long-term MACE-free survival. Limitations include the retrospective design and a requirement for external validation.

## Introduction

Coronary thrombotic events remain a major global health burden, driving urgent efforts to identify high-risk individuals harbouring thin-cap fibroatheroma (TCFA), a critical precursor to acute coronary syndromes.^1,2^ Beyond structural plaque characteristics, emerging evidence highlights macrophages as central mediators in atherosclerotic plaque destabilization. These immune cells actively contribute to plaque vulnerability through the secretion of matrix metalloproteinases (MMPs), which degrade extracellular matrix components, accelerate fibrous cap thinning, and ultimately predispose to rupture.^3^ This mechanistic understanding underscores the clinical value of macrophage imaging as a potential strategy for early risk stratification, enabling targeted interventions in patients with vulnerable plaques at heightened risk of acute myocardial infarction.^4^

Optical coherence tomography (OCT) enables the visualization of macrophages through distinct signal-rich punctate patterns or confluent clusters that exhibit higher reflectivity compared with background speckle noise.^5^ The quantification of macrophages has been explored using a parameter termed the normalized standard deviation (NSD),^6,7^ which has demonstrated significantly increased macrophage density in TCFA cases than in non-TCFA cases,^8^ in culprit than in non-culprit plaques,^7^ and in patients with ST-segment elevation myocardial infarction than in those with stable angina.^9^ Despite these findings, two decades of technological advancement have yet to yield commercial OCT systems equipped with dedicated NSD analysis software. Current NSD applications remain confined to manually preselected regions of interest, predominantly within fibrous caps of fibroatheromas.^6^ Furthermore, the reliability of NSD measurements is compromised by interference from high NSD values associated with other plaque tissue components and by preprocessing steps designed to reduce speckle noise, which may remove macrophage-related information.

Macrophages often form clusters that appear as bands of highly reflective tissues during OCT imaging. *Post hoc* analysis from the CLIMA study showed that a quantitative assessment of the arc of macrophages’ circumferential extension could be obtained with high reproducibility, offering a potentially valuable tool for readily measurable macrophage infiltration.^10^ This metric provides a clinically feasible approach to assess macrophage infiltration burden. Nevertheless, the prognostic implications of macrophage arc quantification remain inadequately characterized, especially regarding its association with long-term major adverse cardiovascular events (MACEs).

## Methods

### Study design

A retrospective, multicentre registry study was undertaken from January 2017 through April 2023, enrolling individuals diagnosed with coronary artery disease (CAD) who underwent coronary angiography alongside OCT at Xinqiao Hospital and Southwest Hospital of the Army Medical University in Chongqing, China. The exclusion criteria included patients with poor OCT image quality, an effective statistical vessel length of <20 mm, clinical data loss, or loss to follow-up.

### Data collection and processing

Data collection adhered to the institutional guidelines and was conducted through the hospital’s electronic medical record system. The SYNTAX score was calculated to assess the coronary anatomy using online calculators at http://www.syntaxscore.com/calculator/start.htm. The OCT data analysis followed strict protocols to ensure accuracy and reliability. OCT was performed based on clinical judgement [e.g. to assess plaque morphology or guide percutaneous coronary intervention (PCI)] in vessels with culprit lesions (consistent with standard clinical practice). When the pre-PCI OCT data were available, they were prioritized for statistical analysis; otherwise, when vascular segments of recently implanted stents were not endothelialized, data from non-stented segments were included. Continuous statistical processing was applied to long vessels that underwent double OCT examinations. The minimum lumen area (MLA) of the target lesion was measured by OCT to quantify coronary lesion burden; the total vessel area and lumen area were measured by OCT, and the plaque area and area stenosis (AS) were calculated to quantify plaque burden. The local ethics board approved the project and was registered in the Chinese Clinical Trial Registry (chictr.org.cn identifier ChiCTR2400091532). The authors assume full responsibility for this study’s design, conduct, and final contents.

### OCT data and analysis

Coronary angiographies were performed using standard techniques, and OCT images were acquired with the ILUMIEN OPTIS system or the OPTIS Mobile system (Abbott, USA) using a non-occlusive technique. OCT image analysis was performed by two experienced investigators (X.H. and X.L.) who were blinded to the clinical presentation; discordance was resolved by a third investigator (W.H.); and there were no borderline results included in the analysis. Only the OCT pullbacks allowed accurate lumen measurement (with a continuous arc of at least 270° around the lumen’s centre), and the qualitative assessment of superficial plaque components was deemed eligible. The study specifically targeted plaques identified by OCT that were surrounded by at least 5 mm of healthy vessels.^2^

### OCT parameter definition

**Figure 1:**
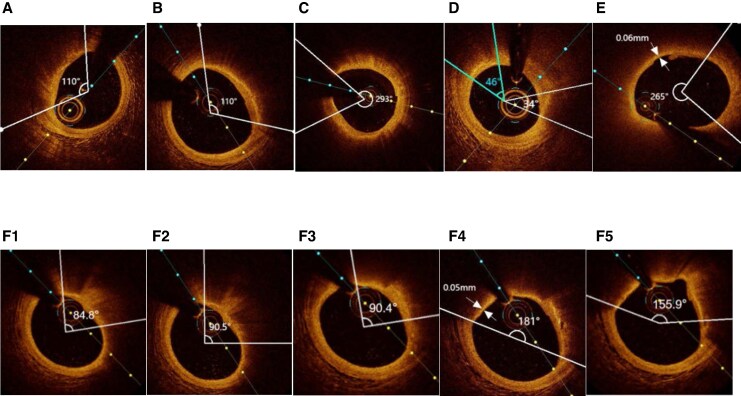
Representative picture of OCT-derived macrophage arc. (*A*) A 110° raster macrophage arc (Grade 3). (*B*) A continuous 110° macrophage arc (Grade 3). (*C*) A large 293° macrophage arc (Grade 4). (*D*) A total angle of 80° by two separated macrophage clusters (Grade 2). (*E*) TCFA (lipid arc 265°, fibre cap thickness 0.06 mm, no macrophage infiltration). (F1 - F5) Sequential frame-by-frame dynamics of macrophages adjacent to a TCFA, showing gradual expansion of the macrophage arc and morphological transition from dot-like to linear structures. In f1–f2, typical macrophages are identified, with intense speckle noise in the background, casting striated shadows. In f3, sharp transverse shadows are projected in the 12–3 o’clock direction, and the rapid frame changes suggest the presence of macrophages. In f4, a TCFA is formed, with a macrophage arc of 181°. In f5, the macrophage arc decreases, and the morphology reverts towards dots form. TCFA, thin-cap fibroatheroma.

The definitions and cut-off values for the OCT parameters were derived from the existing consensus documents and related OCT research findings.^11^ TCFA was defined as having a minimum fibrous cap thickness of <65 μm and lipid core arc extension of >180°.^12^ Macrophages frequently cluster together, forming a band of highly reflective tissue in OCT imaging. The strong attenuating properties of macrophage aggregates cause the casting of a laterally sharp shadow and a rapid change in appearance from frame to frame. These typical characteristics help to differentiate a band of macrophages overlying a fibroatheroma from a true TCFA.^13^ In our OCT imaging, macrophages could be present as dots (single bright spots or clusters of dots without the formation of a clear line) or as lines (confluent accumulations forming a thin bright line). The macrophage arc was measured with the vascular centre as the reference point.^10^ Measurements of the macrophage’s maximum arc were performed at the plaque cross-section that was judged as containing the greatest amount of macrophages.^14^ The mean arc was calculated as the sum of the detectable macrophage arc of each frame divided by the imaged vessel segment (mm). The mean arc score was assessed using a grading scale from 0 to 4 as follows: Grade 0, no macrophage; Grade 1, localized macrophage accumulation; Grade 2, clustered accumulation < 1 quadrant; Grade 3, clustered accumulation ≥ 1 quadrant and <3 quadrants; and Grade 4, clustered accumulation ≥ 3. To distinguish between Grades 1 and 2, the degree of macrophage extension was defined as 30°.^15^ The cumulative grade score was divided by the imaged vessel segment (mm) to calculate the macrophage mean arc score (MAS) (*Figure 1*).

### Follow-up

We conducted a follow-up visit with the participant at 12 months post-discharge and continued follow-up until March 2025. The MACEs included cardiac death and nonfatal myocardial infarction (MI). Cardiac death was defined as any death due to an immediate cardiac cause (e.g. fatal arrhythmia, MI, or heart failure) or unwitnessed death without a non-cardiac explanation. Nonfatal MI was defined as elevation of high-sensitivity cardiac troponin (hs-cTn) ≥ 5 × 99th percentile upper reference limit post-OCT after discharge, accompanied by either new ischaemic symptoms, ECG changes (ST-segment deviation ≥ 1 mm or new pathological Q waves), or imaging evidence of new loss of viable myocardium. Endpoints were assessed by means of dedicated telephone-based interviews and/or office-based direct visits. Events were adjudicated by an independent committee (blinded to OCT findings) to ensure consistency with standardized definitions. Most of the patients were discharged with an indication to dual antiplatelet therapy and optimized lipid-lowering therapy (i.e. maximum tolerated dose) throughout the follow-up period.

### Statistical analysis

Data were described using standard deviations (±SD) for variables with normal or skewed distributions, medians (first–third quartiles) for non-normally distributed measurements, and percentages for categorical variables. Bivariate analyses were conducted using the Student’s *t*-, paired samples *t*-, Mann–Whitney *U*, χ^2^, and Fisher’s exact tests, as appropriate. The receiver operating characteristic (ROC) curve was utilized to evaluate the predictive accuracy of the OCT parameters for plaque risk, with the highest Youden’s index (J) used to determine the optimal cut-off points. Rigorous intra-validation was performed for the macrophage arc-related cut-offs identified by the ROC curve. The 10-fold cross-validation was used to obtain predictive probabilities, and the bootstrap resampling method was combined to construct a ROC curve with a stable 95% confidence interval.

All variables were tested for bivariate association with the primary clinical endpoint and if nominally significant (*P* < 0.05) were simultaneously forced into a hierarchical multivariate Cox regression model to identify independent OCT outcome predictors and to calculate their adjusted hazard ratio (HR). The Schoenfeld residual test was used to verify that the model satisfied the proportional hazards assumption. All analyses were performed using SPSS version 24.0 (IBM, Armonk, NY, USA).

## Results

### Demographic and procedural data

From January 2017 to April 2023, coronary OCT was evaluated in 1307 patients and 1436 vessels. Ultimately, a total of 1025 patients with 1173 blood vessels were included in the analysis; among the 1173 vessels, 919 (80.8%) were pre-PCI segments (analysed before stenting), and 254 (19.2%) were non-stented segments from vessels after PCI or with recently implanted stents (analysed post-stenting but with incomplete endothelialization). Macrophages were detected in 79.9% of the vessels. Quantitative analysis revealed a median maximum arc of 134°, a median mean arc of 51.39°, and a median mean arc score (MAS) of 1.68. Clinical and procedural baseline characteristics of the study population are detailed in *Table 1*. Interobserver reproducibility for macrophage arc measurements, evaluated via intraclass correlation coefficient (Cronbach’s alpha), demonstrated strong agreement with a coefficient value of 0.956. Based on the estimation results of single measures, the intraclass correlation coefficient used to assess the intra-observer reproducibility of this study was 0.989 (*P* < 0.001).

**Table 1:** Univariable and multivariable Cox regression analysis of patients’ characteristics and OCT findings associated with MACEs

	Population (1025)	Patients with clinical events (61)	Patients without clinical events (964)	Univariate HR (95% CI)	Multivariate HR (95% CI)
Age (years)	61 (53–69)	58 (52–69)	61 (54–69)	0.991 (0.969–1.014) *P* = 0.443	N/A
Female gender (%)	344 (33.5)	17 (27.9)	327 (33.9)	0.889 (0.507–1.556) *P* = 0.679	N/A
LVEF (%)	63 (59–67)	60 (56–66)	63(59–67)	0.976 (0.953–1.000) *P* = 0.050	0.994 (0.962–1.018) *P* = 0.476
Hypertension (%)	638 (62.2)	41 (67.2)	597 (61.9)	1.562 (0.915–2.666) *P* = 0.099	N/A
Hypercholesterolaemia (%)	312 (30.4)	25 (41)	287 (29.7)	1.904 (1.142–3.175) *P* = 0.014	1.894 (1.085–3.304) *P* = 0.025
Smoking habit (%)	635 (61.9)	40 (65.6)	595 (61.7)	1.432 (0.844–2.429) *P* = 0.183	N/A
Diabetes (%)	339 (33.1)	26 (42.6)	313 (32.4)	1.860 (1.119–3.090) *P* = 0.017	2.142 (1.227–3.738) *P* = 0.037
Prior MI (%)	159 (15.5)	14 (23)	145 (15.0)	1.845 (1.016–3.351) *P* = 0.044	N/A
Prior PCI (%)	249 (24.3)	17 (27.9)	232 (24.1)	1.439 (0.821–2.523) *P* = 0.204	N/A
SYNTAX score	5.0 (2.0–8.0)	7.0 (2.75–11.25)	5.0 (2.0–8.0)	1.017 (0.999–1.035) *P* = 0.063	N/A
MLA (mm^2^)	2.42(1.64–3.57)	2.0(1.45–2.89)	2.68(1.62–3.41)	0.827(0.686–0.996) *P* = 0.034	0.831(0.557–0.998) *P* = 0.023
AS (%)	69.7(57–78.5)	73.65(68.35–82.15)	70.4(58.9–78.9)	1.028(1.008–1.048) *P* = 0.005	1.028(0.984–1.064) *P* = 0.259
Blood sugar (mmol/L)	5.5 (5.0–6.6)	5.7 (5.0–6.7)	5.5 (5.0–6.6)	1.063 (0.953–1.185) *P* = 0.273	N/A
Blood sugar ≥ 7 mmol/L	211 (19.7)	13 (21.3)	198 (19.5)	1.298 (0.699–2.412) *P* = 0.409	N/A
LDL-C (mmol/L)	2.1 (1.6–2.7)	2.0 (1.3–2.7)	2.1 (1.6–2.7)	0.908 (0.665–1.238) *P* = 0.540	N/A
LDL-C ≥ 2.6 mmol/L	294 (26.8)	16 (26.2)	278 (26.8)	1.059 (0.595–1.884) *P* = 0.846	N/A
*OCT macrophage finding*	Vessels (1173)				
*Mean arc (°)	51.39 (22.08–105.04)	136.37 (77.37–214.78)	47.45 (20.69–97.69)	1.293 (1.221–1.368) *P* < 0.0001	1.312 (1.188–1.449) *P* < 0.0001
Mean arc ≥ 97.88°	275 (23.4)	40 (65.5)	235 (24.4)	7.628 (4.267–13.636) *P* < 0.0001	4.379 (2.162–8.868) *P* < 0.0001
*MAS	1.68 (0.82–3.07)	3.47 (2.36–5.30)	1.54 (0.77–2.90)	1.473 (1.338–1.621) *P* < 0.0001	1.434 (1.235–1.665) *P* < 0.0001
MAS ≥ 2.27	378 (32.2)	44 (72.1)	334 (34.6)	6.704 (3.539–12.700) *P* < 0.0001	3.729 (1.752–7.939) *P* < 0.0001
*Maximum arc (°)	134 (90–203)	234 (167.25–298.25)	131 (88.25–198)	1.441 (1.287–1.547) *P* < 0.0001	1.275 (1.137–1.429) *P* < 0.0001
Maximum arc ≥ 157.5°	402 (34.3)	45 (73.8)	357 (37.0)	6.902 (3.569–13.349) *P* < 0.0001	2.952 (1.409–6.185) *P* < 0.0001
*Plaque characteristics*					
TCFA (%)	199 (17.0)	35 (57.4)	164 (17.0)	7.457 (4.484–12.404) *P* < 0.0001	2.720 (1.394–5.306) *P* = 0.001
Plaque rupture (%)	201 (17.1)	26 (42.6)	175 (18.2)	3.566 (2.146–5.926) *P* < 0.0001	1.491 (0.778–3.587) *P* = 0.129
Presence of mean arc ≥ 97.88° + TCFA (%)	129 (11.0)	29 (47.5)	100 (10.4)	8.779 (5.296–14.552) *P* < 0.0001	6.343 (3.619–11.117) *P* < 0.0001
Presence of MAS ≥ 2.27 + TCFA (%)	153 (13.0)	30 (49.2)	123 (12.8)	7.509 (4.537–12.428) *P* < 0.0001	5.604 (3.204–9.802) *P* < 0.0001
Presence of maximum arc ≥ 157.5° + TCFA (%)	141 (13.0)	31 (50.8)	110 (11.4)	8.149 (4.924–13.487) *P* < 0.0001	5.957 (2.821–8.711) *P* < 0.0001

LDL, low-density lipoprotein; LVEF, left ventricular ejection fraction; MACE, major adverse cardiovascular event; MAS, mean arc score; MI, myocardial infarction; N/A, not applicable, variables excluded from multivariate analysis due to non-significant univariate P values; OCT, optical coherence tomography; PCI, percutaneous coronary intervention; TCFA, thin-cap fibroatheroma.

*, each macrophage parameter was individually included in distinct multivariate models rather than combined in a single model.

During the longitudinal follow-up period, which extended up to 98 months, 104 patients (9.2%) were lost to follow-up. The median follow-up duration was 33 months (interquartile range: 23–46 months), during which 61 patients (5.9%) experienced MACEs. Stratified analysis showed that among 199 patients with TCFA and 826 non-TCFA patients, the adjusted MACE incidences were 17.6% and 3.14%, respectively.

### Clinical predictors

Patients who experienced MACEs demonstrated significant associations with several clinical and imaging parameters in univariate Cox regression analysis: diabetes (*P* = 0.017, HR = 1.860, 95% CI: 1.119–3.090), hypertension (*P* = 0.099, HR = 1.562, 95% CI: 0.915–2.666), left ventricular ejection fraction (LVEF; *P* = 0.050, HR = 0.976, 95% CI: 0.953–0.993), hypercholesterolaemia (*P* = 0.014, HR = 1.904, 95% CI: 1.142–3.175), TCFA (*P* < 0.001, HR = 7.457, 95% CI: 4.484–12.404), mean arc (per 30° increase, HR = 1.293, 95% CI: 1.221–1.368, *P* < 0.001), MAS (HR = 1.473, 95% CI: 1.338–1.621, *P* < 0.001), and maximum arc (per 30° increase, HR = 1.441, 95% CI: 1.287–1.547, *P* < 0.001). After adjusting for significant clinical characteristics in multivariate Cox regression analysis, TCFA (HR = 5.094, 95% CI: 2.385–10.879; *P* < 0.001), diabetes (HR = 1.972, 95% CI: 1.113–3.495; *P* = 0.020), hypercholesterolaemia (HR = 1.930, 95% CI: 1.069–3.485; *P* = 0.029), mean arc (per 30° increase, HR = 1.312, 95% CI: 1.188–1.449; *P* < 0.001), MAS (HR = 1.434, 95% CI: 1.235–1.665; *P* < 0.001), and maximum arc (per 30° increase, HR = 1.275, 95% CI: 1.137–1.429; *P* < 0.001) remained independent predictors of MACEs (*Table 1*).

**Figure 2:**
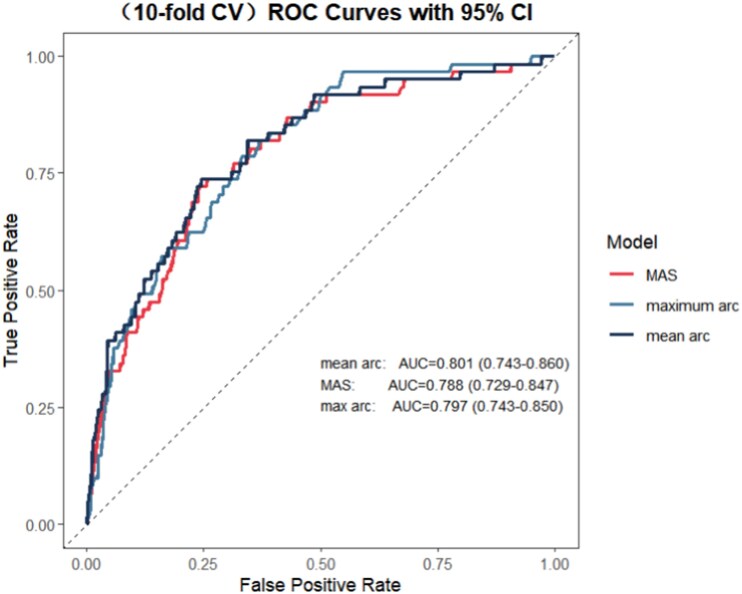
ROC curve analysis to predict MACEs. MACE, major adverse cardiovascular event; ROC, receiver operating characteristic.

To determine optimal cut-off values for macrophage-related features in predicting MACEs, ROC curve analysis was performed. Intra-validation results showed that the predictive efficacy of all cut-offs remained stable, with AUC ranging from 0.729 to 0.860 and all Youden’s indices > 0.5(*Figure 2*). The ROC-derived thresholds—maximum arc ≥ 157.5°, mean arc ≥ 97.88°, and MAS ≥ 2.27 (*Table 2*; Supplementary data)—were then utilized in Kaplan–Meier estimation of cumulative MACE incidence to facilitate further survival analysis.

**Table 2:** Best cut-off value and AUC of ROC curves

Variables	Best cut-off value	AUC	Youden’s index	Sensitivity	Specificity	*P* value
Mean arc	97.88	0.801	0.534	78.2%	75.2%	<0.001
MAS	2.27	0.788	0.521	87.3%	64.8%	<0.001
Maximum arc	157.5	0.797	0.504	87.3%	63.1%	<0.001

AUC, area under the curve; MAS, mean arc score.

### Predictor effect of macrophage arc

**Figure 3:**
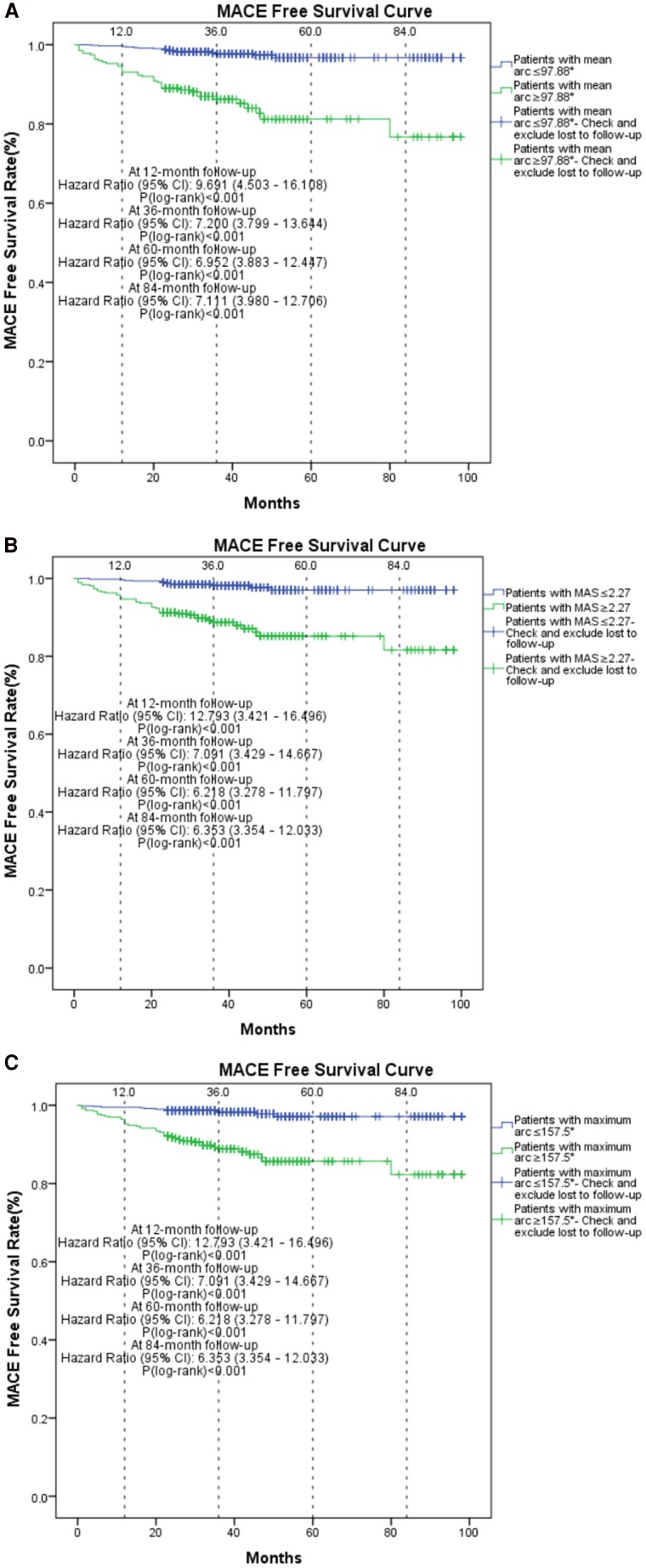
MACE-free curves on the basis of the presence of mean arc (*A*), MAS (*B*), and maximum arc (*C*) are shown. There were significant differences in cumulative rates of MACE at 12-, 36-, 60-, and 84-month follow-up between the groups. The cumulative rates of MACEs were higher in the large arc group than in the small arc group (*P* < 0.001). MACE, major adverse cardiovascular event.

The Kaplan–Meier analysis revealed a significant increase of MACEs in the group with mean arc ≥ 97.88° (HR = 7.628, 95% CI: 4.267–13.636, *P* < 0.0001). Similar significant associations were observed for MAS ≥ 2.27 (HR = 6.704, 95% CI: 3.539–12.700, *P* < 0.0001) and maximum arc ≥ 157.5° (HR = 6.902, 95% CI: 3.569–13.349, *P* < 0.0001). When comparing follow-up time points, the disparity in the cumulative incidence of MACE events was most pronounced at the 12-month assessment, as illustrated in *Figure 3*.

### Combination predictor effect of macrophage arc and TCFA

The simultaneous presence of TCFA and mean arc ≥ 97.88° was significantly more frequent in experiencing the MACEs at the multivariate Cox regression analysis (HR = 6.343, 95% CI: 3.619–11.117, *P* < 0.001), as well as the combination of TCFA and MAS ≥ 2.27 (HR = 5.604, 95% CI: 3.204–9.802, *P* < 0.001) and TCFA and maximum arc ≥ 157.5° (HR = 5.957, 95% CI: 2.821–8.711, *P* < 0.001).

### Independent predictor of macrophage arc

In the subgroup of patients without TCFA, a mean arc ≥ 97.88° (HR = 6.685, 95% CI: 2.751–16.246, *P* < 0.001), MAS ≥ 2.27 (HR = 5.126, 95% CI: 2.042–12.867, *P* < 0.001), and maximum arc ≥ 157.5° (HR = 4.490, 95% CI: 1.788–11.278, *P* = 0.001) were associated with the main composite endpoint at the multivariate Cox regression analysis.

## Discussion

This multicentre and large-scale study introduces macrophage arc as novel and easily quantifiable OCT-derived indicators of adverse outcomes. Specifically, the findings showed that (i) the macrophage arc values (maximum arc ≥ 157.5°, mean arc ≥ 97.88°, and MAS ≥ 2.27) were predictors of MACEs, (ii) combining macrophage arc parameters with TCFA further enhanced predictive power for MACEs, and (iii) notably, in TCFA-negative patients, macrophage arc parameters exhibited an independent predictive capacity for MACEs.

### Comparison with previous macrophage assessment methods

Most previous OCT studies classified macrophages using a binary (presence or absence) definition,^16,17^ which only merely identifies macrophages but provides an incomplete assessment of inflammatory burden and cannot reflect the extent or magnitude of infiltration. Some subsequent studies adopted semi-quantitative grading systems, such as those by Taguchi *et al.*^18^ and Vergallo *et al.*^19^ Although these systems offer a meaningful improvement over binary classification, they remain semi-quantitative and subject to subjective variability, thereby limiting precise and continuous quantification. Furthermore, Liu *et al.*^20^ recently proposed a cut-off value of 72° for mean macrophage arc, which was the median value obtained by only measuring the maximum extension of macrophages in the plaque segment in a single-centre small-sample cohort. However, our large-sample, multicentre, long-term follow-up study assessed global inflammatory burden across the entire vessel segment and established thresholds directly based on their association with MACE endpoints.

### Macrophage maximum arc and prognosis

The role of local macrophages in the progression of plaque vulnerability has been demonstrated in both animal models and human studies.^21,22^ Specifically, culprit lesions in non-ST-elevation acute coronary syndrome (NSTE-ACS) patients (*n* = 32) exhibit significantly increased macrophage density, characterized by a higher maximum arc,^14^ which can be rapidly visualized and quantitatively evaluated via OCT. However, the prognostic implications of these macrophage distribution patterns remain underexplored. Our study demonstrates that the macrophage maximum arc serves as a robust predictor of MACEs, thereby providing a valuable tool for guiding real-time assessment and therapeutic decision-making in daily clinical practice.

### Macrophage mean arc/MAS and prognosis

The findings of multi-focal inflammation^23^ and simultaneous plaque rupture^24^ in patients with ACS have expanded the concept of vulnerable plaques into a broader conceptual framework of the ‘vulnerable vessel’.^25,26^ Consequently, the measurement of macrophage filtration in the target segment is clinically important for multi-focal inflammation evaluation.

Automated OCT pullback typically captures the coronary segments of varying lengths for analysis. Adjusting the accumulated macrophage arc relative to the imagined vessel length (mm) may better reflect the mean level of inflammation within a target segment, facilitating comparison across different observation ranges. In this study, the mean arc and MAS were developed to quantitatively evaluate the macrophage arc and confirm the important role of macrophage arc parameters in predicting cardiovascular outcomes. With a 31.2% increase in risk for every 30° increase in macrophage mean arc (HR = 1.312 per 30°), these findings demonstrate a clear dose–response relationship between the degree of macrophage infiltration and the risk of MACE. For lesions with high macrophage arc (e.g. mean arc ≥ 97.88°), intensified optimal medical therapy (OMT)—including high-intensity statins, PCSK9 inhibitors, or anti-inflammatory agents (e.g. colchicine)—may be prioritized to stabilize inflammation, given the link between macrophage activity and plaque vulnerability.

### Combination predictor effect of macrophage arc and TCFA

TCFA, characterized by a fibrous cap < 65 μm and a large lipid core, is prone to rupture or erosion, triggering platelet aggregation and thrombus formation, which may lead to MI or cardiac death. However, inflammation-driven MMP activity, such as macrophage infiltration, weakens the fibrous cap, exacerbating TCFA instability. In this study, we identified macrophage arc measurements and TCFA as independent predictors of MACEs. Importantly, combining macrophage arc analysis with TCFA assessment yielded a synergistic prognostic value (*P* < 0.0001). Macrophage arc is a high-risk feature of plaque inflammation. Structural abnormalities combined with inflammatory activation jointly aggravate plaque instability. The combination of the two realizes dual risk stratification of ‘structure + inflammation’, thus having a synergistic predictive value.

### Independent predictor of macrophage arc

Post-mortem pathological studies have established that the majority of ACS events arise from the TCFA; however, approximately 25% of cases^27^ result from endothelial erosion without plaque rupture. The reason may be attributed to pro-thrombotic effects of macrophages by promoting plaque surface erosion through sustained endothelial inflammation (via IL-6 and TNF-α secretion) and direct activation of platelets by tissue factor.^28^ These mechanisms highlight macrophages’ intrinsic ability to destabilize plaque integrity and amplify thrombotic risk without requiring TCFA-associated rupture. In this study, we demonstrate for the first time that macrophage arc measurements serve as robust independent predictors of adverse cardiovascular outcomes in non-TCFA populations. Specifically, multivariable regression analysis identified macrophage arc metrics—with HRs exceeding 6 for mean arc as significant determinants of MACEs. Most of the patients in this study received Dual Antiplatelet Therapy and statin therapy, which inhibited plaque inflammation and macrophage activity. Under this condition, macrophage arc was still a strong independent predictor of MACE (all HR > 3), which highlights the clinical value of this index even more—even under standard drug therapy, high macrophage arc still indicates high risk in patients and requires intensive intervention.

Our research also possesses several strengths. First, it is the multicentre, large-sample nature of our study. Second, we introduced novel macrophage arc measurements. Third, multiple prognostic effects are evaluated.

### Limitations

The present study has several limitations. First, given its retrospective design, the study is inherently susceptible to selection bias, the current results have not been validated in an independent external cohort, and the retrospective nature may also introduce bias due to loss to follow-up during the long-term study period, resulting in a relatively small number of MACE events. Second, we only performed OCT examination on the target vessel, which represents segmental macrophage infiltration rather than the inflammatory level of multiple coronary arteries. Third, this is a long-term study, so repeat angiography was not performed on every patient who experienced MACEs. Consequently, it is impossible to determine whether the MACEs are caused by the target vessel or target lesion, such as TCFA. Fourth, this study detected macrophages, not in a direct histopathological way. Although measuring the macrophage arc is not a real quantitative assessment of macrophage infiltration, the simplicity of commercial OCT-defined processing algorithms may provide researchers and clinicians with a valuable tool. Medication adherence was assessed by telephone and patient self-report, which carries subjective reporting bias and was not adjusted in the model. Future prospective studies should adopt objective records, such as pharmacy refills, to validate adherence and minimize confounding.

### Conclusion

In conclusion, macrophage arc parameters are strongly associated with long-term cardiovascular risk, indicating novel and easily quantifiable OCT-derived indicators of adverse outcomes. These findings highlight that future studies should validate their potential for clinical decision-making and prognostic assessment.

## Supplementary Material

qyag107_Supplementary_Data

## Data Availability

All original clinical and imaging data generated in this study are available from the corresponding authors upon reasonable request.

## Lead Author Biography

Xiaohui Zhao, MD, Department of Geriatrics, Xinqiao Hospital, Army Medical University, Chongqing, China. Email: zhaoxiaohui@tmmu.edu.cn

## Clinical perspectives

Macrophage arc could refine risk stratification in patients with CAD, particularly those with non-TCFA plaques (where traditional markers are limited). In clinical practice, high macrophage arc may prompt intensified risk factor management (e.g. stricter lipid control and anti-inflammatory therapy) or closer surveillance. For interventionalists, it may guide lesion selection for PCI (e.g. prioritizing lesions with high arc and haemodynamic significance). Prospective trials are needed to test these applications.
