# Supplementary material for: Optical coherence tomography-derived macrophage arc as a novel biomarker for predicting adverse cardiovascular events in coronary artery disease: a multicentre study
Source: Eur Heart J Imaging Methods Pract. 2026 Jun 12;4(1):qyag107. doi: 10.1093/ehjimp/qyag107 (PMC13312119; doi:10.1093/ehjimp/qyag107)

Supplementary

bootstrap calibration curve and decision curve analysis


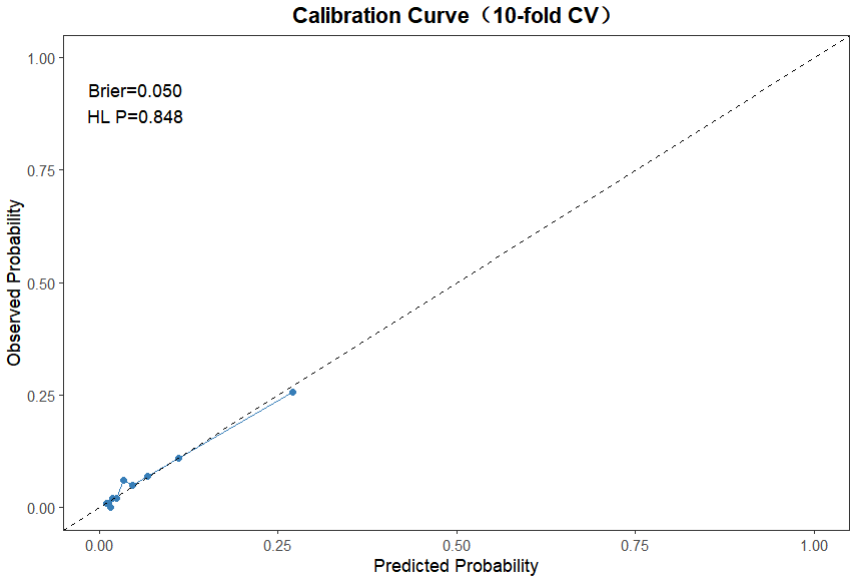

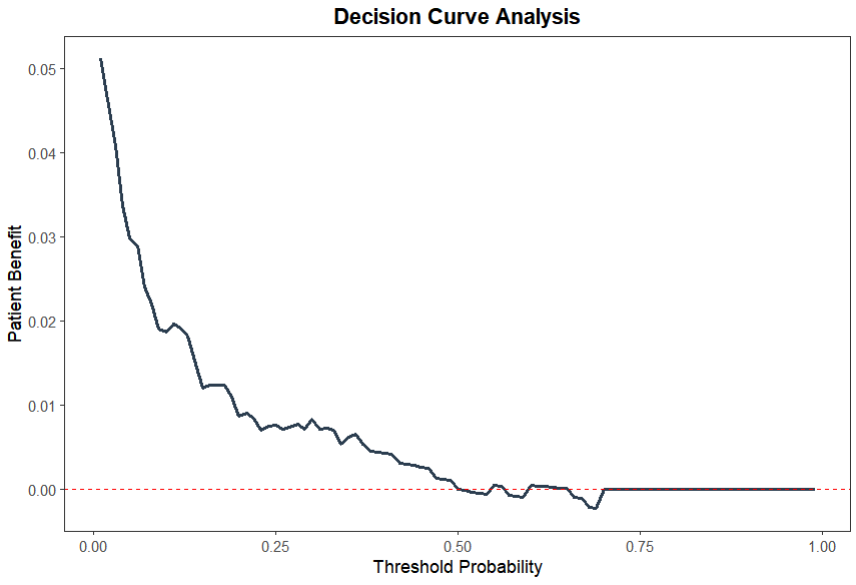


Schoenfeld residual test

LVEF


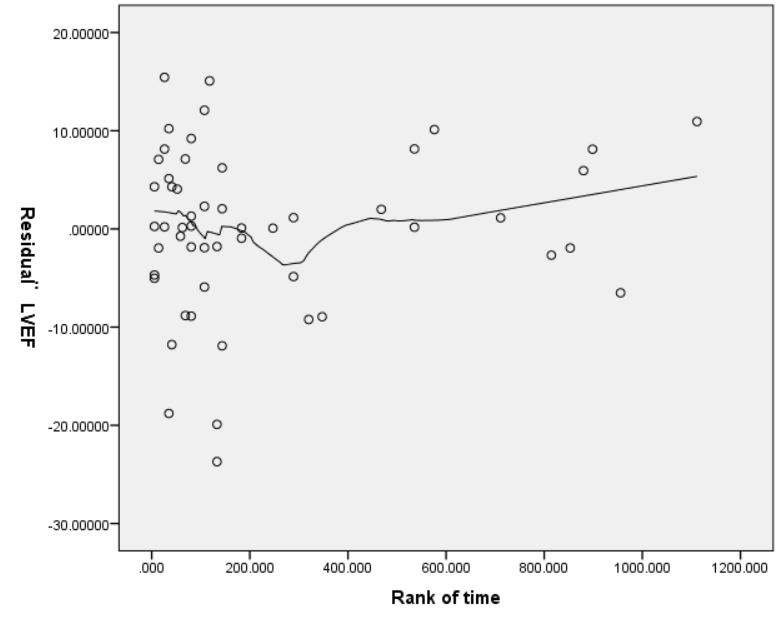


Hypercholesterolemia


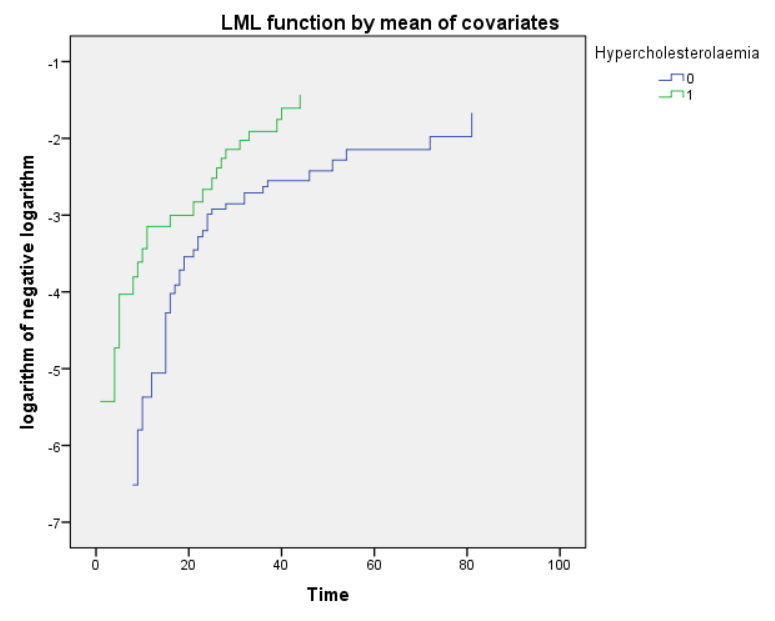


Diabete


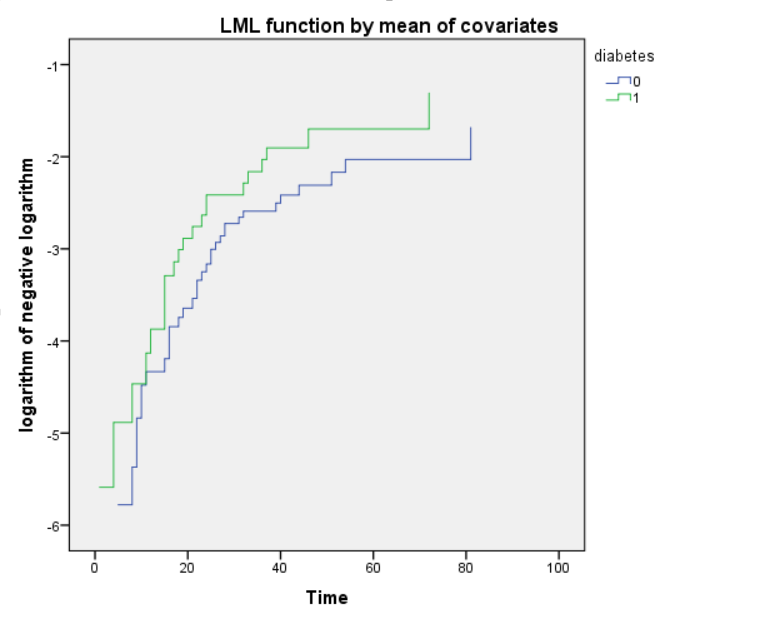


MLA


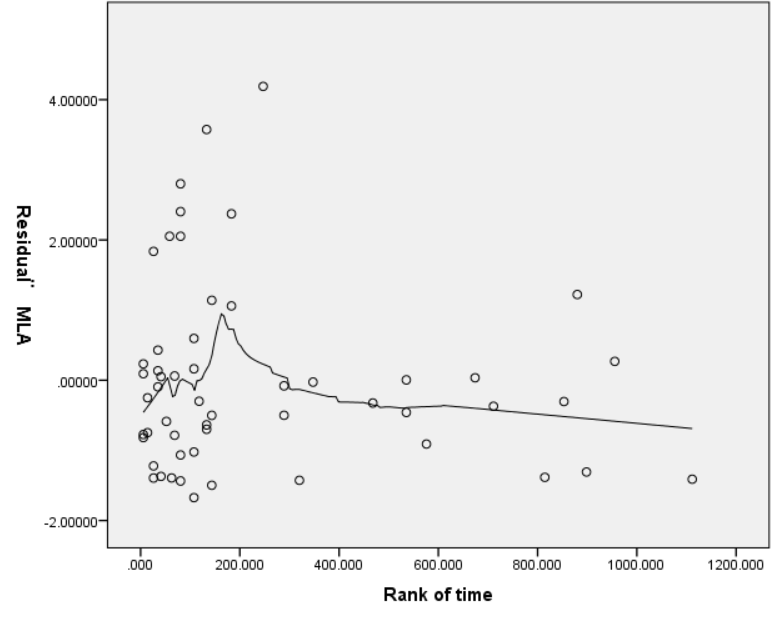


AS


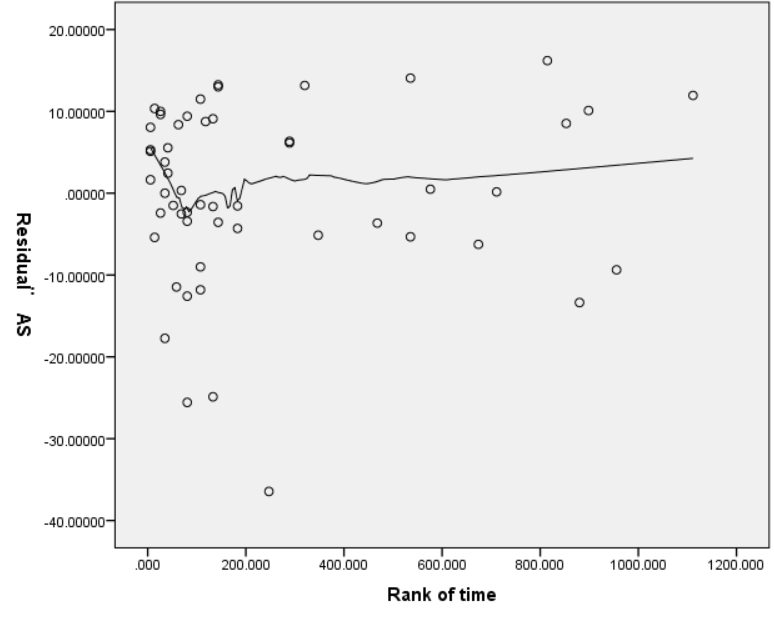


Mean arc


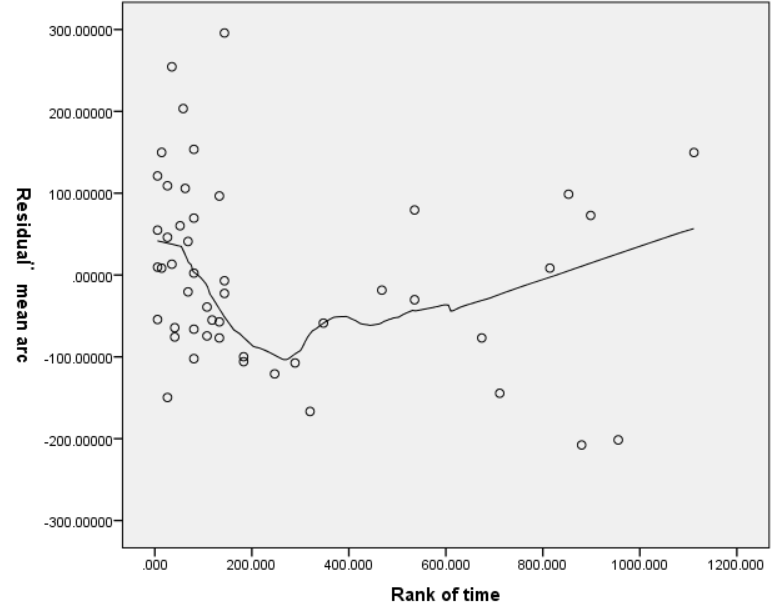


MAS


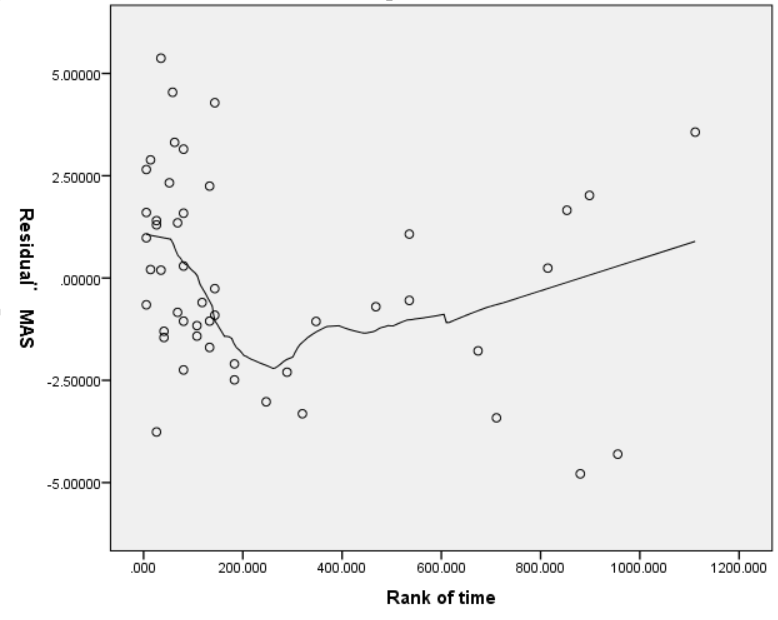


Maximum arc


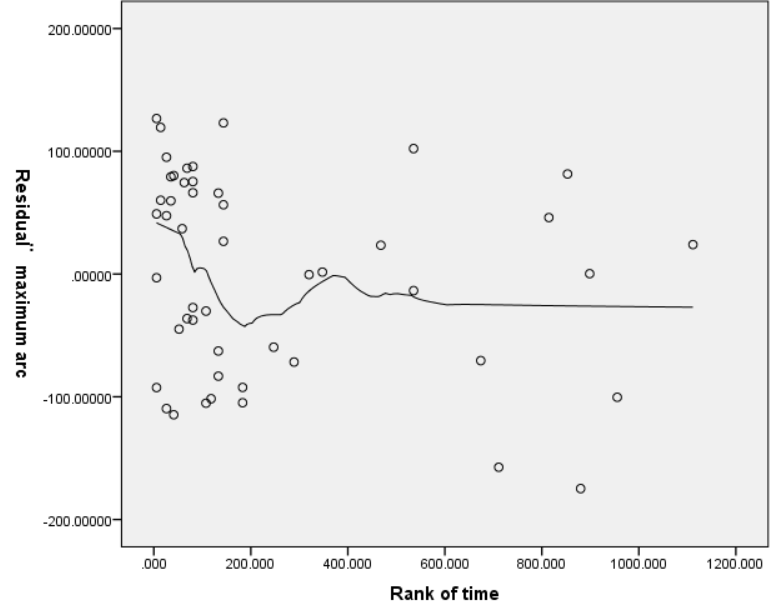

Supplement: qyag107_Supplementary_Data [file qyag107_supplementary_data.docx]
